# Intradialytic optical assessment of C-mannosyl tryptophan removal using spent dialysate

**DOI:** 10.1038/s41598-025-01844-z

**Published:** 2025-06-12

**Authors:** Joosep Paats, Annika Adoberg, Liisi Leis, Jürgen Arund, Kai Lauri, Merike Luman, Risto Tanner, Jana Holmar, Kristjan Pilt, Ivo Fridolin

**Affiliations:** 1 Department of Health Technologies, Tallinn University of Technology, 19086 Tallinn, Estonia; 2 Centre of Nephrology, North Estonia Medical Centre, Sütiste Tee 19, 13419 Tallinn, Estonia; 3 SYNLAB Eesti OÜ, Veerenni 53a, 10138 Tallinn, Estonia

**Keywords:** C-mannosyl tryptophan, C-glycosyltryptophan, Hemodialysis, End stage kidney disease, Uremic solutes, Spectrophotometric analysis, Biomarkers, Nephrology

## Abstract

C-mannosyl tryptophan (CMW), also known as C-glycosyltryptophan, is a novel biomarker that is strongly correlated to chronic kidney disease (CKD) incidence and progression risk and mortality among earlier stages of CKD patients prior to end stage kidney disease. This study determined concentrations of CMW in blood and spent dialysate of CKD patients on chronic hemodialysis (HD) for the first time, and investigated the possibility for optical estimation of CMW concentrations in spent dialysate, its intradialytic removal and time-averaged concentration (TAC) of CMW based on optical measurements of spent dialysate. In total, 264 pre- and postdialysis blood samples, and 528 spent dialysate samples from 88 HD sessions of 22 patients were analyzed using high pressure liquid chromatography and spectrophotometry. We identified that CMW concentrations in CKD patients on chronic HD are over 10 times higher compared to earlier reported CMW concentrations in healthy subjects. The concentration of CMW in spent dialysate can be monitored based on spectrophotometric analysis of spent dialysate (r > 0.939, standard error: 0.07 μmol/L) and it is possible to evaluate CMW-based HD adequacy parameters, such as reduction ratio, mass of total removed solute, and TAC without blood sampling. In future, optical monitoring of CMW could be potentially used to improve clinical management of hemodialysis patients.

**Supplementary Information:**

The online version contains supplementary material available at 10.1038/s41598-025-01844-z.

## Introduction

Chronic kidney disease (CKD) affects approximately 10% of the global population^1^. CKD is a progressive disease, and if uncontrolled, CKD can progress to end stage kidney disease (ESKD). In case of ESKD, kidney replacement therapy (KRT) is required to prevent death from uremic syndrome, caused mainly by uremic solutes that accumulate to the body in ESKD^2^. The most applied form of KRT is hemodialysis (HD), accounting for approximately 69% of all forms of KRT that were applied to treatment of 3.9 million ESKD patients in 2017^3^. During HD metabolic waste products, termed as uremic solutes, and excessive water are removed from ESKD patients’ bodies, and balance of electrolytes is restored. By the year 2015, over 270 different uremic solutes had been identified of which many are considered to exert toxic effect based on experimental or observational studies and randomized controlled trials^4,5^. Since retention of uremic solutes in CKD arises from impaired kidney function through decreased filtration, secretion, reabsorption, generation or metabolic breakdown of solutes, efforts have been taken to identify reliable metabolite biomarker for diagnostic purposes and for improving health outcomes of CKD^6–9^. However, as biomarker levels can be influenced by anthropometric parameters, nutritional status, gut microbiome in addition to impaired kidney function and etiology^9^, it can be challenging to characterize status of patients from different populations and stages of CKD based on the concentration of individual metabolite, such as in case of using creatinine for glomerular filtration rate (GFR) estimation^6,9–11^.

Among uremic solutes^5^, monomeric C-mannosyl tryptophan (CMW) with monoisotopic molecular weight of 366.14 Da, also known as C-glycosyltryptophan, is a clinically promising biomarker that has been shown to be independently and strongly correlated with health outcomes and estimated GFR (eGFR) in healthy subjects and patients in different stages of CKD from populations of European and non-European ancestry independent of age^12–15^. Particularly, CMW is a degradation product of C-mannosylatated proteins^16^, which have undergone a unique post-translational modification that features attachment of a single α-mannose to the tryptophan residue of a substrate protein via C–C bond, the only known form of protein C-linked glycosylation in humans^17–19^. In contrast to the O-glycosidic and N-glycosidic bonds, C-glycosyl bond that connects the carbohydrate to target molecule is generally resistant to hydrolysis and exhibits more metabolic stability^20^. Specifically, C-mannosylation is mediated by C-mannosyltransferases in endoplasmic reticulum that transfer a mannose from dolichol phosphate mannose usually to the first tryptophan in the motif Trp-Xaa-Xaa-Trp of a substrate proteins^18,21,22^. So far, approximately 30 different C-mannosylated proteins, mostly from thrombospondin type I repeat superfamily and cytokine receptor type I family, have been experimentally verified in humans^18^. However, 18% of all human secreted or transmembrane proteins have been predicted to be C-mannosylated^21^, which appears to be essential for proper folding, stability, transportation, and function of the C-mannosylated proteins^18^. Eventually, during the proteolytic degradation of C-mannosylated proteins, a monomeric CMW is generated that is thought to be not further catabolized in the body^16,18^. Consequently, CMW is excreted from the body via urine^23,24^, reportedly with the clearance of CMW close to that of inulin^25^.

Concerning clinical relevance, several researchers from different workgroups have shown that blood concentration of CMW is a good indicator of eGFR^12,25,26^ and a predictor of eGFR decline^12,13,27^. A recently published study of Burgh et al., which enrolled over 4800 participants, found that among the total of 1381 studied metabolites, CMW was the most strongly associated metabolite with CKD risk and progression^13^. While serum concentration of CMW increases exponentially with the decline of eGFR^25^, urine CMW levels are also altered with CKD and provide information about CKD outcomes similarly^14^. In a metabolome-wide association study enrolling 5087 CKD patients, with measurements of 1487 metabolites in urine, higher levels of CMW in urine were significantly associated with all of the adverse outcomes of interest, such as progression to ESKD, a combined progression to ESKD and acute kidney injury, and death^14^. Notably, out of the 55 identified urine metabolites that were significantly associated with at least one of the endpoints in that study, CMW was the only metabolite related to all of the examined outcomes, disregarding the unknown metabolite X-12117^14^. Moreover, some studies have shown that concentration of CMW is more strongly correlated to measured GFR compared to established serum creatinine–based parameters, regardless of the age and muscle mass of the subject^12,25,26^.

Although CMW is a clinically promising biomarker and an endogenous uremic solute with potential intrinsic toxicity, the dialytic removal characteristics of CMW and the relationship between clinical outcomes and CMW have not been assessed so far for ESKD patients who are receiving HD. Additionally, biochemical methods have not been developed to determine CMW concentrations yet, and quantification of CMW with mass spectrometry or chromatographical methods remains time consuming and expensive.

The aim of this study was to determine the levels of CMW in ESKD patients’ blood and spent dialysate using high pressure liquid chromatography (HPLC), and investigate the possibility for optical estimation of CMW concentrations in spent dialysate, and assess time-averaged concentration (TAC) of CMW and its intradialytic removal as reduction ratio (RR) and total removed solute (TRS) based on spectrophotometric analysis of spent dialysate without blood sampling.

## Methods

### Ethics

The study protocol was approved by the Tallinn Medical Research Ethics Committee at the National Institute for Health Development in Estonia (decision no. 2205). A written informed consent was obtained from all patients involved in the study and the study was conducted in accordance with the Declaration of Helsinki.

### Subjects and hemodialysis treatments settings

Twenty-two ESKD patients on chronic HD treatment (thrice weekly) were enrolled into the study from the Centre of Nephrology, North Estonia Medical Centre, Estonia as part of the multicenter OLDIAS2—Online Dialysis Sensor Phase 2 project, encompassing patients from diverse European countries^28^. All patients fulfilled the following criteria: over 18 years old with vascular access (arteriovenous fistula/ arteriovenous graft) capable of managing blood flow of at least 300 mL/min, without clinical signs of infection or other active acute clinical complications and an estimated life expectancy over 6 months. Of the patients, 17 were male and 5 were female, with an average age (± SD) of 55 ± 17 years, who had spKt/Vurea of 1.47 (1.23–1.67), given as median (interquartile range), in routine treatment. Detailed description of demographic and clinical data of patients has been presented in Table 1.

**Table 1:** Clinical data of the 22 end stage kidney disease (ESKD) patients monitored during total of 88 hemodialysis sessions.

Entity of the Data	Specifications
Cause of ESKD	Diabetes (4); Hypertension (8); Glomerulonephritis (3); Tubulointerstitial nephritis (3); Renal carcinoma (2); Other (2)
Age [years]	55 ± 17
Gender	M 17; F 5
Race, Caucasian [%]	100
BMI [kg/m2]	26.8 ± 5.8
BW [kg]	81.5 ± 21.3
Ultrafiltration volume [mL]	2565 ± 1190
Urinary volume [mL]	0 [14 patients]; 700 (335–825) [8 patients]
Serum total protein [g/L]	62.8 ± 5.5
Hematocrit [%]	34.4 ± 3.5
Serum calcium [mmol/L]	2.25 ± 0.16
Serum phosphorus [mmol/L]	1.92 (1.63–2.29)
Serum parathyroid hormone [pmol/L]	28.7 (16.8–41.9)
Vascular access	AVF 15; AVG 7
Dialysis vintage [months]	23 (11–83)
spKt/Vurea	1.47 (1.23–1.67)

Numerical values are given as a mean ± standard deviation or as a median and interquartile range (Q1–Q3). M, male; F, female; BMI, body mass index; BW, body weight; spKt/V, single-pool criterion of the dose of dialysis; AVF, arteriovenous fistula, AVG, arteriovenous graft.

During the study period, each patient received four midweek dialysis sessions with predefined settings (Table 2) to vary dialytic removal of uremic toxins using Fresenius 5008 dialysis machine (Fresenius Medical Care, Bad Homburg v. d. Höfe, Germany). On the remaining days of the week, patients received their standard treatment regimen as prescribed in their routine clinical care. The predefined HD settings included: one low flux hemodialysis with minimal settings and three different post-dilution haemodiafiltration (HDF) treatments with the prescribed duration of 4 hours. Three different dialyzers with polysulphone-based membranes: Xevonta® Lo15 (B. Braun Medical, Melsungen, Germany), Helixone® FX800 and Helixone® FX1000 (Fresenius Medical Care, Bad Homburg v. d. Höfe, Germany) with effective membrane areas of 1.5 m^2^, 1.8 m^2^ and 2.2 m^2^ were used, respectively. Treatment settings were kept constant during each dialysis session: the applied blood flow ranged from 200 to 400 mL/min and dialysate flow from 300 to 800 mL/min, respectively. Substitution volume over all HDF sessions varied from 11.7 to 31.2 L per session. In the end of each treatment, the effective average treatment settings of each session were recorded from the screen of dialysis machine (Table 2) for the following calculations.

**Table 2:** The effective dialysis treatment settings of the predefined hemodiafiltration (HDF) and hemodialysis (HD) sessions.

Entity of the Data	Standard HDF	Low Flux HD	Medium HDF	High HDF
Effective blood flow, mL/min (Qb)	296 (295–297)	199 (198–199)	297 (296–298)	368 (356–377)
Effective dialysate flow, mL/min (Qd)	359 (355–493)	297 (297–298)	789 (788–795)	791 (788–796)
Substitution volume (Vs, L)	22.0 (20.0–23.0)	0	14.9 (14.9–15)	25.2 (23–27.9)
Substitution rate, mL/min	95.5 (87–99)	0	66 (65–66)	112 (100–123)
Ultrafiltration rate, mL/min	9.0 (6.3–14.6)	12.6 (8.3–16.4)	11.6 (6.3–13.8)	11.25 (7.5–16.7)
Dialyzer model^a^	FX800; FX1000	Lo15	FX1000	FX1000
Number of dialyses (N)	22	22	22	22

Standard HDF marks treatment settings that were prescribed to the patients in routine clinical care. Numerical values of each modality are given as median and interquartile range (Q1–Q3).

^a^Specification of dialyzers and effective membrane areas: Helixone® FX800 1.8 m^2^, Helixone® FX1000 2.2 m^2^ (Fresenius Medical Care, Bad Homburg v. d. Höfe, Germany), Xevonta® Lo 15 1.8 m^2^ (B. Braun Medical, Melsungen, Germany).

### Sample collection and analysis

During each treatment session, blood samples were collected from the arterial blood line and spent dialysate samples from the drain outlet tube of HD machine at discrete sampling times.

Blood samples were drawn immediately before the start, and immediately at the end of the dialysis session using the slow pump method, i.e. blood flow was decreased to 50 mL/min 2 min prior of the sampling to avoid any effects of recirculation. Additionally, one blood sample was taken 30 min postdialysis. Blood samples were collected into 3.5 mL and 5 mL Becton Dickinson Vacutainer SST II Advance (Franklin Lakes, NJ, USA) tubes, kept still for 30 min to allow clotting and were thereafter centrifuged for 20 min with swing-out buckets at 3000 g. After centrifugation, serum was separated from the blood cells and subjected to further analyses.

Spent dialysate samples were collected from the drain outlet of the dialysis machine 7, 60, 120, 180 min after the start, and at the end of dialysis session (240 min) before initiating the slow pump method. Additionally, the total spent dialysate from the whole procedure was collected to a large tank. After the procedure, the tank was weighted with DE 300K50DL platform scale (Kern & Sohn GmbH, Balingen, Germany) and after carefully mixing, a sample was taken from the tank to estimate mass of total removed solute. All dialysate samples were first collected into 120 mL Becton Dickinson Vacutainer urine collection cups (Franklin Lakes, NJ, USA) and aliquoted afterwards into 5 mL Brand cryogenic tubes (Brand GMBH & CO KG, Wertheim, Germany) for analytical chemistry analyses.

A total of 264 blood samples (including 176 pre- and immediate postdialysis, and 88 blood samples taken 30 min postdialysis) and 528 spent dialysate samples of 22 patients from 88 different dialysis sessions were collected and subjected to further analysis on the same day. One set of serum samples was sent to the clinical chemistry laboratory (SYNLAB Eesti OÜ, Tallinn, Estonia) to determine concentration of most used dialysis dose marker molecule urea in serum using standardized methods. Furthermore, a second set of samples were analyzed in analytical chemistry laboratory of Tallinn University of Technology to determine concentration of CMW in the collected serum and dialysate samples using HPLC.

Prior to HPLC analysis serum samples were filtered by centrifugation using Sartorius Vivacon 30 kDa cut-off filters (Göttingen, Germany) at 14.000 g for 3 h at 37 °C. Before serum filtration, cut-off filters were washed through with 400 µL type I ultrapure water (Millipore Synergy UV, Burlington, MA, USA) by centrifugation at 14.000 g for 15 min at 37 °C. To stabilize uric acid to undissociated form, 1 µL of formic acid (Sigma-Adrich, St. Louis, MO, USA) was added to the serum filtrate and likewise 10 µL of formic acid was added to unprocessed spent dialysate samples before HPLC analysis.

The HPLC analysis was carried out with Ultimate 3000 Series HPLC system from Dionex, a division of Thermo Scientific company (Sunnyvale, CA, USA) equipped with a quaternary gradient pump unit (DGP-3600RS), a thermostated autosampler (WPS-3000TSL analytical), a column oven (TCC-3000RS), a diode array spectrophotometric detector (DAD-3000RS), and a fluorescence detector (FLD-3400RS). Separation was performed using two continuous columns of Poroshell 120 C18 4.6 × 150 mm with a security guard Poroshell 120 C18 4.6 × 5 mm from Agilent Instruments (Santa Clara, CA, United States) with the temperature of columns at 40 °C and autosampler at 4 °C. A three-step linear gradient elution program was employed with the total flow rate of 0.6 mL/min as described earlier^29^. The eluent consisted of a mixture of 0.05 M formic acid adjusted to pH 4.25 with ammonium hydroxide (A), and organic solvent of HPLC grade methanol and HPLC-S grade acetonitrile, both from Honeywell (Charlotte, NC, USA) in ratio of 9:1 containing 0.05 M ammonium formiate salt (B). The obtained chromatographic data were processed with Chromeleon 7.1 software by Thermo Scientific (Waltham, MA, USA).

Chromatographic peak of CMW on the serum and the spent dialysate samples’ chromatograms was identified based on the comparison of the retention time and fluorescence spectra with those of the standard solution of CMW and further confirmed by the MS/MS mass spectra of the peak specific to CMW^24,30^. Mass spectra were registered with a quadrupole time-of-flight mass spectrometer micrOTOF-Q II with an electrospray ionization (ESI) source (Bruker, Billerica, USA) that was coupled to HPLC system. Mass spectrometer was used in negative ion mode and the operating conditions were as follows: mass range of m/z 50–700; ion source temperature of 200 °C, ESI voltage of 4.5 kV, ESI nebulization gas flow of 8.0 L/min, drying gas flow of 1.2 bar, detector voltage of 2.03 kV, acquisition rate of 1 Hz as described earlier by Arund et al.^29^. Mass calibration was carried out using a solution of sodium formate (10 mmol/L) in the range of m/z 50 to 700. Data were acquired with Compass HyStar (version 3.2) and processed with Compass DataAnalysis (version 4.0 SP1) software (both Bruker, Billerica, USA).

Aqueous calibration standard solutions of CMW were prepared from reference substance purchased from Toronto Research Chemicals Inc (Toronto, Ontario, Canada) with purity of assay > 98% (thin layer chromatography) diluted in type I ultrapure water (Millipore Synergy UV, Burlington, MA, USA) and followed by treatment in ultrasonic bath for 30 min to ensure complete dissolution of the compound. The standard solutions with different known concentrations were analyzed with the same HPLC method as the serum and the spent dialysate samples to calibrate the HPLC system for determination of CMW concentration based on the acquired fluorescence signals (excitation: 280 nm; emission 360 nm). The linearity of the fluorescence signal was investigated in the concentration range of 0.017–10.5 µmol/L with 6 points (3 replicate injections each) achieving Pearson correlation coefficient of > 0.999 and inter-day precision (relative standard deviation) < 0.26% over a given range.

In addition, an extra set of spent dialysate samples were analyzed separately with ultraviolet (UV)- and fluorescence spectroscopy using UV-3600 spectrophotometer and RF-6000 spectrofluorometer, both from Shimadzu (Kyoto, Japan). UV absorption spectra were recorded over a wavelength range of 200–400 nm with sampling interval of 1 nm (spectral bandwidth of 2 nm) and quartz cuvette with optical path length of 5 mm using pure dialysis solution as a reference. Fluorescence spectra were measured over the excitation wavelength range of 200–400 nm with the increment of 10 nm and the emission wavelength range of 210–600 nm with the increment of 1 nm at room temperature. A quartz cuvette with an optical path length of 4 mm were used and bandwidths of both excitation and emission monochromators were set to 5 nm. Emission spectra were corrected for inner filtering of the excitation beam as described earlier based on the measured absorbance values at excitation wavelength^31,32^. Thereafter, the emission spectra were smoothed using moving average filter with the window size of 10.

In parallel to spent dialysate sampling, on-line fluorescence and UV absorption measurements were carried out during each dialysis session in real-time with an optical sensor prototype (Optofluid Technologies OÜ, Tallinn, Estonia) connected to the drain outlet of the dialysis machine. Measurement results of the sensor prototype were compared to laboratory results to detect possible errors related to the sample drawing during the self-tests of the HD machine^33^ or incorrect sample labelling. As a result, the data of one serum and 33 spent dialysate samples were excluded during the data preprocessing from the following analysis due to aforementioned errors.

### Data analysis and CMW removal evaluation

Forward stepwise regression was used to create a model for predicting concentration of CMW in spent dialysate samples using data of UV absorption and fluorescence of dialysate samples. For this purpose, the patients’ data were equally partitioned to a calibration and validation datasets based on the patients’ pseudonymised identification numbers, which were used to develop and validate the model, respectively. The exclusion of samples’ data due to the self-tests of haemodialysis machine resulted in final sizes of datasets for calibration N = 244 and validation N = 251 as the excluded datapoints were not equally distributed. The fluorescence intensity with excitation in range of 240–400 nm and UV absorbance at a single wavelength (based on linear regression analysis between concentration of CMW and UV absorbance in spent dialysate samples of calibration dataset) were included as predictor variables of training data. The upper limit of the selectable variables was limited to three during model training to avoid overfitting. During the model training, a variable was included into the model in case the *p*-value was less than 0.05 for an F-test of the change in the sum of squared error that resulted from adding the variable.

Subsequently, the accuracy of the created model was evaluated on the calibration and validation data using Bland Altman^34^ and regression analysis. Systematic error (BIAS) was calculated for the models as follows:

$$BIAS = \frac{{\mathop \sum \nolimits_{i = 1}^{N} e_{i} }}{N}$$

where e_i_ is the i-th residual (difference between HPLC determined and modelled CMW) and N is the number of observations^35^.

Standard error of performance corrected for BIAS was calculated for the model as follows^35^.

$$SE = \sqrt {\frac{{\mathop \sum \nolimits_{i = 1}^{N} \left( {e_{i} - BIAS} \right)^{2} }}{N - 1}}$$

In order to assess removal of CMW during HD sessions, dialysis adequacy parameters, such as RR, TRS and TAC were estimated. The parameters were calculated based on HPLC determined CMW concentrations in serum and spent dialysate samples, and optical model-predicted CMW concentrations in spent dialysate samples to evaluate possibility of estimating these parameters non-invasively.

The RR of CMW during each dialysis session was calculated based on serum samples as follows:

1 $$\begin{array}{*{20}c} {RR_{{\text{ serum}}} = \frac{{C_{{{\text{pre}}}} {-} C_{{{\text{post}}}} }}{{C_{{{\text{pre}}}} }} \times 100\% } \\ \end{array} ,$$

where C_pre_ and C_post_ are HPLC determined pre- and immediate postdialysis concentrations of CMW in serum samples, respectively. Moreover, effective RR was additionally calculated using serum samples taken predialysis and 30 min postdialysis to assess extent of rebound effect caused by postdialysis redistribution of CMW in body compartments^36^. Furthermore, RR was similarly found for urea to compare removal dynamics of CMW with kinetic behavior of urea.

To calculate spent dialysate-based RR of CMW, concentrations of CMW in spent dialysate samples determined with HPLC or estimated with optics-based model were used:

2 $$\begin{array}{*{20}c} {RR_{{\text{ dialysate}}} = \frac{{D_{7} {-} D_{240} }}{{D_{7} }} \times 100\% ,} \\ \end{array}$$

where D_7_ and D_240_ are the concentrations of CMW in spent dialysate samples taken at 7 min after the start and at the end (240 min) of the dialysis procedure, respectively.

The TRS of CMW was calculated based on total dialysate collection as follows:

3 $$\begin{array}{*{20}c} {TRS = D_{{\text{T}}} \cdot W_{{\text{T}}} } \\ \end{array} ,$$

where *D*_T_ is the concentration of CMW in the dialysate collection tank determined with HPLC or estimated with optics-based model and W_T_ is the weight of the dialysate collection tank. The density of spent dialysate was assumed to be 1 kg/L.

To compare concentrations of CMW in serum and spent dialysate directly, spent dialysate concentrations of CMW (*D*_*t*_) were normalized by spent dialysate and blood flow rates (*D*_*t* norm_), thereby roughly compensating for dialyzer clearance^37,38^:

4 $$\begin{array}{*{20}c} {D_{{t{\text{ norm}}}} = D_{t} \cdot \frac{{Q{\text{d}} + Q{\text{subs}} + UF }}{{Q{\text{b}}}}} \\ \end{array} ,$$

where *Q*_b_ is effective blood flow and the numerator “dialysate flow rate (*Q*_d_) + substitution rate (*Q*subs) + ultrafiltration rate (*UF*)” marks the total flow rate of spent dialysate.

TAC of CMW in blood over dialysis sessions was calculated from HPLC determined CMW concentrations as the logarithmic mean concentration, as proposed by Lim et al.^39^:

5 $$\begin{array}{*{20}c} {TAC = \frac{{(C_{{{\text{pre}}}} - C_{{{\text{post}}}} )}}{{\ln \left( {\frac{{C_{{{\text{pre}}}} }}{{C_{{{\text{post}}}} }}} \right)}}} \\ \end{array} ,$$

where C_pre_ and C_post_ are pre- and immediate postdialysis concentrations of CMW determined with HPLC, respectively.

In parallel, TAC of CMW was estimated (TAC_opt_) from optical model-predicted CMW spent dialysate concentrations:

6 $$\begin{array}{*{20}c} {TAC_{{{\text{opt}}}} = a \cdot \frac{{(D_{{7{\text{ norm}}}} - D_{{240{\text{ norm}}}} )}}{{\ln \left( {\frac{{D_{{7{\text{ norm}}}} }}{{D_{{240{\text{ norm}}}} }}} \right)}},} \\ \end{array}$$

where *D*_7 norm_ and *D*_240 norm_ mark optically estimated CMW concentrations in spent dialysate samples taken 7 and 240 min after the start of dialysis, respectively, which have been normalized by spent dialysate and blood flow rates (Table 2) according to Eq. 4. The regression coefficient “*a*” was determined based on calibration dataset.

All data analysis was done using MATLAB R2020b (MathWorks, Natick, MA, USA) software. Individual differences of serum and dialysate based dialysis adequacy parameters were compared using Bland Altman analysis^34^. The Anderson–Darling test was employed to assess the normality of the datasets, which determined the appropriate statistical tests for subsequent analysis. For normally distributed data, Student’s two-tailed paired t-test was used to compare differences between related samples from the same patient, while the unpaired two-tailed t-test was applied to compare differences between treatment modalities. For non-normally distributed data, the Wilcoxon test was utilized. In all statistical tests, a *p*-value of < 0.05 was considered significant.

## Results

**Fig. 1:**
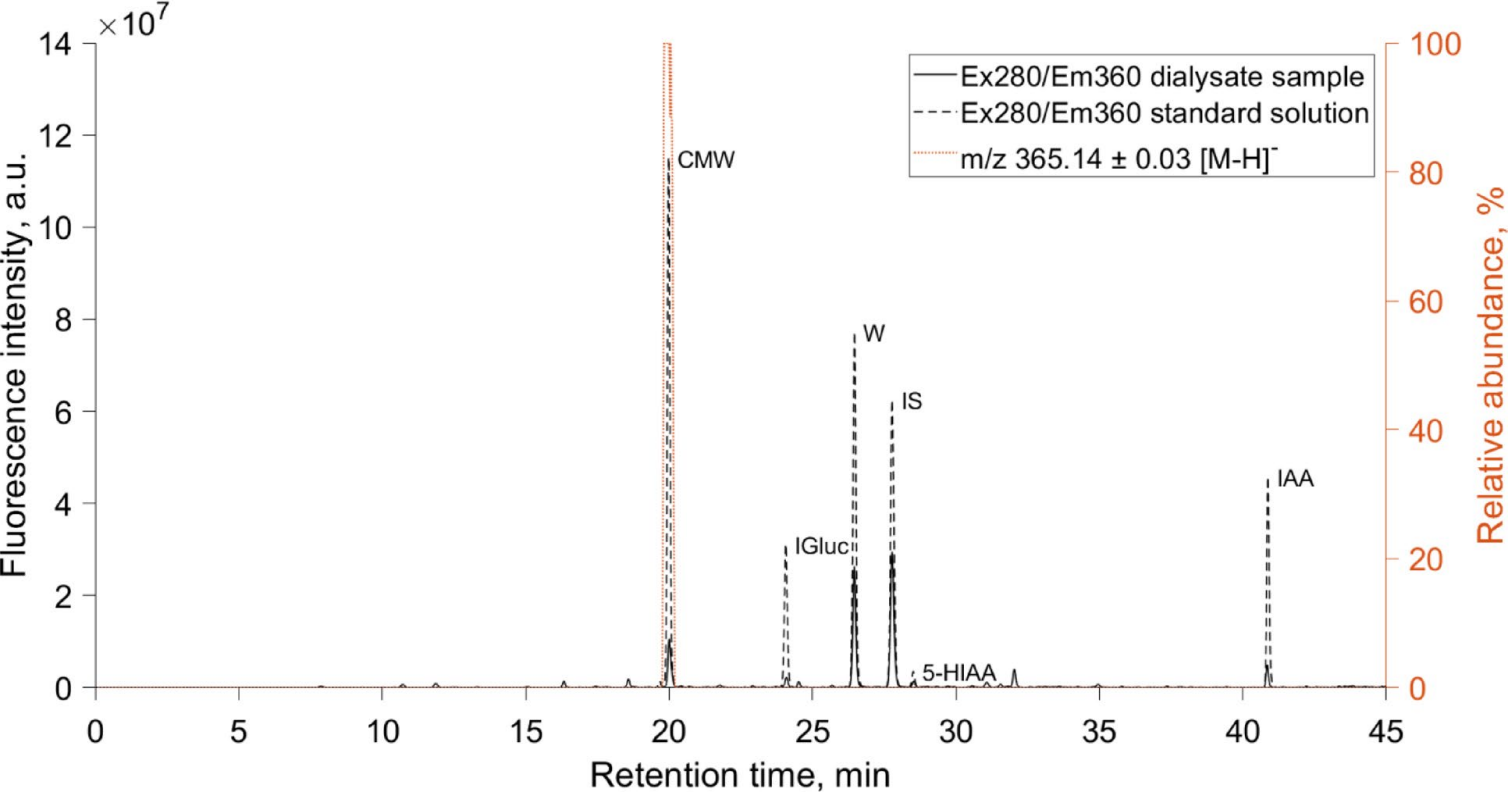
A characteristic chromatogram of a spent dialysate sample and superimposed chromatogram of a standard solution, including C-mannosyl tryptophan (CMW), indoxyl glucuronide (IGluc), tryptophan (W), indoxyl sulfate (IS), 5-hydroxyindole-3-acetic acid (5-HIAA), indole-3-acetic acid (IAA). Extracted ion chromatogram of a spent dialysate sample in negative ion mode characteristic for CMW (m/z⁻^1^ 365.14 ± 0.03) is marked as (…), and fluorescence signals at excitation (Ex) 280 nm/Emission (Em) 360 nm are marked as (–) for dialysate sample, and as (--) for the standard solution.

In total, 264 serum samples and 528 spent dialysate samples were analyzed with HPLC. Figure 1 shows an example of a characteristic chromatogram of a spent dialysate sample and overlaid chromatogram of a standard solution, including reference substances.

The fluorescence and relative abundance peak of ion m/z⁻^1^ 365.14 ± 0.03 with retention time of 20.01 min on the spent dialysate chromatogram was identified as a CMW based on the mass spectrum (Supplementary Fig. S1), fluorescence spectrum and the retention time of the peak that were identical to the standard solution of CMW. The concentration of CMW in the serum and spent dialysate samples was quantified based on the acquired fluorescence signals as described in the Methods section. The HPLC analysis showed that the median (interquartile range) predialysis concentration of CMW in serum samples was 2.78 (2.20–3.22) μmol/L and 0.52 (0.38–0.73) μmol/L at the end of treatment, and in spent dialysate samples 0.64 (0.52–0.79) μmol/L and 0.13 (0.08–0.22) μmol/L, respectively.

**Fig. 2:**
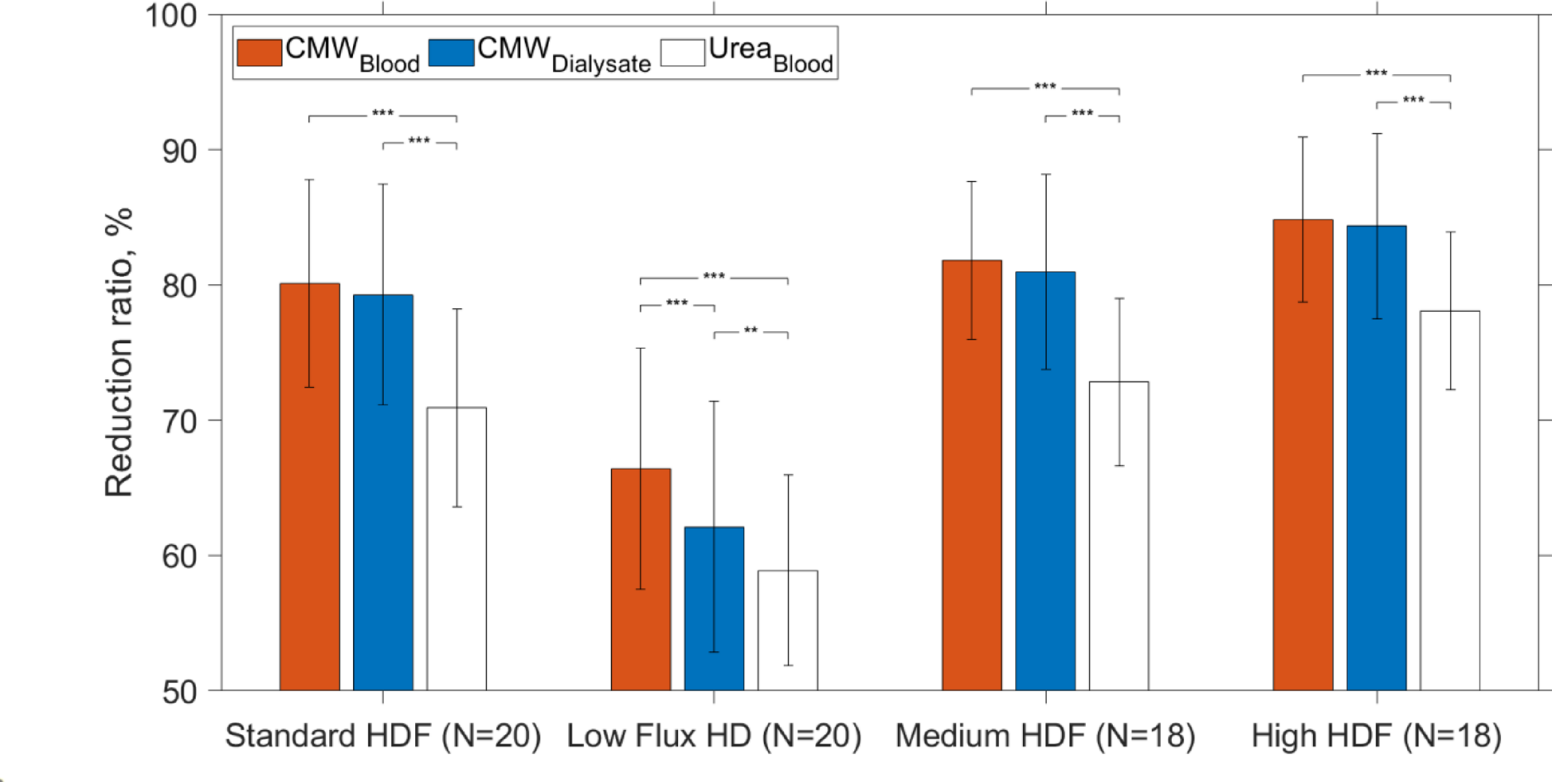
Reduction ratios (average ± SD) of C-mannosyl tryptophan (CMW) and urea for hemodialysis (HD) and different hemodiafiltration (HDF) modalities (see Table 2 in Methods section) evaluated based on serum and spent dialysate samples collected from the start and end (240 min) of the dialysis sessions; statistical intra-modality differences, evaluated using paired two-tailed t-tests, are denoted as follows: *** (*p* < 0.001), ** (*p* < 0.01), * (*p* < 0.05).

Figure 2 compares average RRs of CMW and urea during dialysis sessions calculated from serum and spent dialysate concentrations for different dialysis modalities and settings.

It can be seen from the Fig. 2 that the RR of CMW is higher in comparison with RR of urea (> 6.7 percentage points) for all modalities (*p* < 0.001) and that the removal efficacy of CMW and urea are similarly affected by the treatment settings, calculated based on serum samples. What stands out is that the RRs of both solutes are significantly higher (*p* < 0.001) for most efficient HD modality (High HDF) in comparison with Low Flux HD with RR (± SD) of 84.8 ± 6.1% vs 66.4 ± 8.9% for CMW and RR (± SD) of 78.1 ± 5.8% vs 58.9 ± 7.0% for urea (see Supplementary Fig. S2 for inter-modality statistical differences). Whereas the differences between RRs of HDF sessions are smaller. A comparison of the RR results of CMW calculated based on serum and spent dialysate samples showed that values found from spent dialysate samples were slightly lower (4.3 percentage points) than serum-based values for Low Flux HD sessions with relatively low blood and dialysate flow rates (*p* < 0.001), but not for HDF sessions (*p* > 0.15). The median (interquartile range) RR over all sessions based on predialysis and immediately taken postdialysis serum sample was 80.1 (72.3–86.0)% for CMW, and for urea 71.3 (63.0–78.0)%, respectively. The rebound effect significantly affected the RRs of both solutes (*p* < 0.01). Namely, effective RR, which was calculated based on predialysis and 30 min postdialysis serum samples was lower: 74.7 (66.6–81.2)% for CMW and 66.2 (58.2–73.3)% for urea. Furthermore, the extent of rebound effect was not statistically different between CMW and urea (*p* = 0.530).

**Fig. 3:**
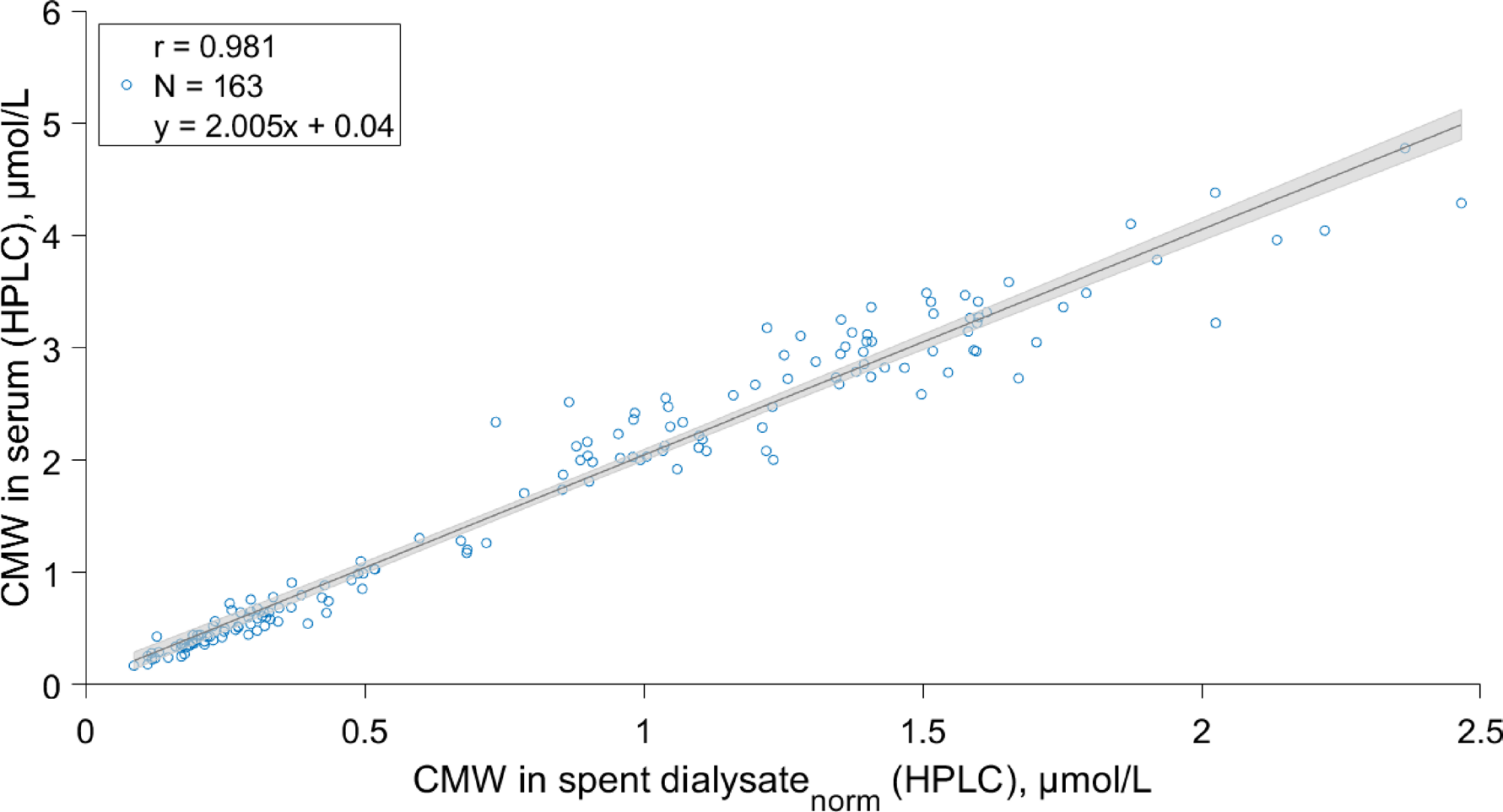
Correlation between C-mannosyl tryptophan (CMW) concentration determined with high pressure liquid chromatography (HPLC) in serum and CMW in spent dialysate normalized (norm) by spent dialysate flow rate and effective blood flow rate based on Eq. 4 (see Methods section). Black continuous line indicates the regression line and grey area indicates the 95% CI of the slope.

Figure 3 shows a scatter plot of the relationship between the concentration of CMW in serum and CMW concentration in spent dialysate samples normalized by blood and spent dialysate flow rates using Eq. 4; with exclusion of 13 paired spent dialysate datapoints due to the HD machines’ self-tests from the total of 176 pre- and postdialysis blood samples. It is evident from the figure that blood and spent dialysate concentrations of CMW are strongly correlated after normalization by treatment settings (r = 0.981, *p* < 0.001).

**Fig. 4:**
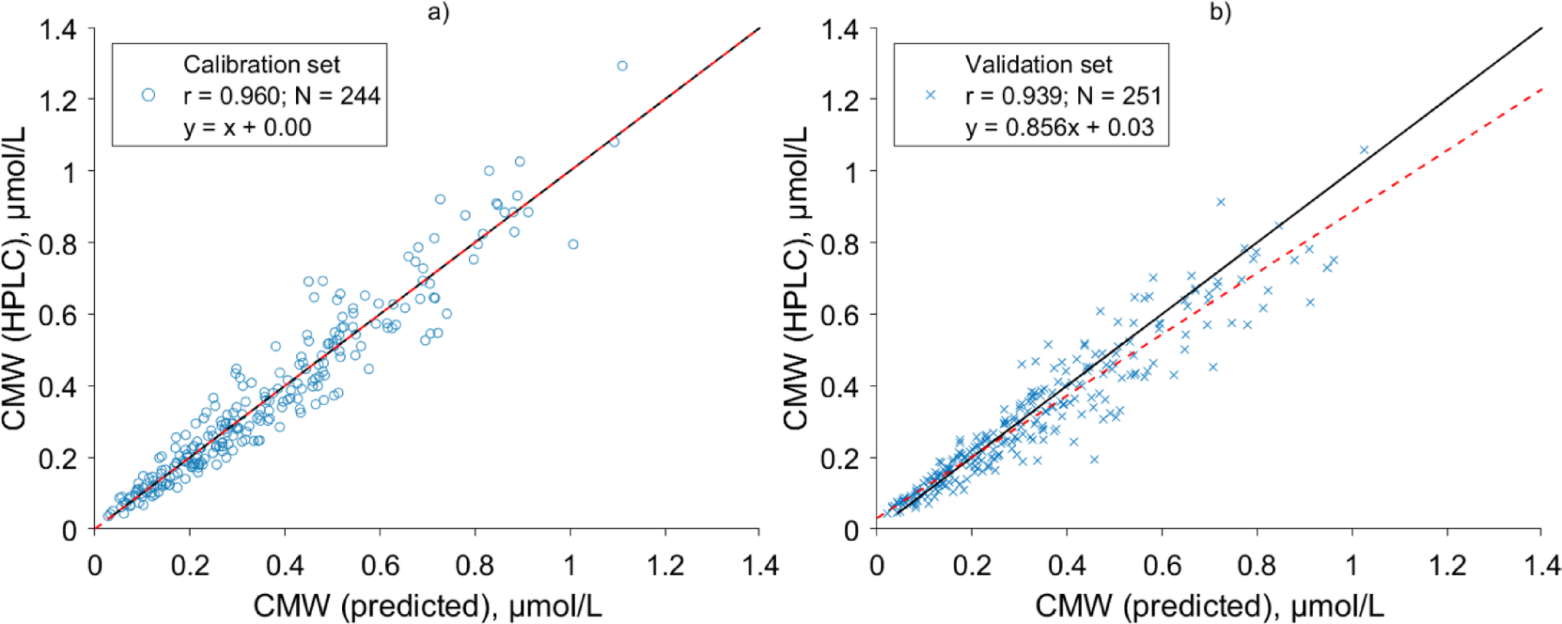
Scatter plots of the relationship of C-mannosyl tryptophan (CMW) concentration in spent dialysate determined with high pressure liquid chromatography (HPLC) and estimated with optics-based predictive model for (**a**) calibration set and (**b**) validation set. Black continuous line indicates the identity line and dashed red line marks the regression line.

In addition to fluorescence data at excitation from 240 to 400 nm, UV absorbance at 295 nm was included as predictor variable of model training data, based on linear regression analysis between concentration of CMW and UV absorbance in spent dialysate samples (Supplementary figure S3). The linear regression model that was created based on calibration dataset to estimate concentration of CMW in spent dialysate from the optical spent dialysate measurements included Ex250Em380 and Ex260Em349, and UV absorbance at 295 nm as variables with the highest predictive power. The scatter plots of the calibration and the validation groups comparing CMW determined with HPLC and predicted based on the optical measurements are shown on Fig. 4.

The model yielded high accuracy (BIAS ± SE) on both calibration and validation set. Pearson correlation coefficient of 0.960 (*p* < 0.001) with the accuracy of 0.00 ± 0.07 μmol/L was achieved for the calibration group (Fig. 4a), and Pearson correlation coefficient of 0.939 (*p* < 0.001) with the accuracy of − 0.02 ± 0.07 μmol/L was achieved for the validation group (Fig. 4b) between the CMW concentration in spent dialysate determined with HPLC and the corresponding values predicted using the model, respectively.

Table 3 compares the HD performance parameters, such as RR, TRS and TAC of CMW evaluated based on HPLC analysis of serum or spent dialysate samples and using output from the optics-based predictive model (Fig. 4) for all data; with exclusion of 14 RR dialysate, 16 RR serum; 16 TAC and 2 TRS values, which were removed prior data analysis due to the HD machines’ self-tests and incorrect sample labelling.

**Table 3:** Hemodialysis performance parameters for all modalities combined based on reduction ratio (RR), total removed solute (TRS) and time averaged concentration (TAC) of C-mannosyl tryptophan (CMW).

Clinical parameter	CMW HPLC median (Q1-Q3)	CMW Opt median (Q1-Q3)	*p*-value	Accuracy (BIAS ± SE)	Pearson correlation coefficient
RR _dialysate_ (%, N = 74)	78.7 (68.6–85.7)	78.5 (67.0–84.6)	0.811	0.7 ± 3.7	0.958
RR _serum_ (%, N = 72)	80.3 (72.3–85.9)	77.8 (66.1–84.5)	0.347	2.4 ± 4.9	0.939
TRS (μmol, N = 86)	40.25 (34.29–47.89)	43.84 (36.75–52.12)	0.213	− 1.72 ± 7.95	0.792
TAC (μmol/L, N = 72)	1.34 (1.13–1.54)	1.39 (1.17–1.59)	0.442	0.00 ± 0.25	0.717

Results based on high pressure liquid chromatography (HPLC) analysis of serum or spent dialysate samples were compared with the results calculated from the output of the optical (Opt) model using Wilcoxson test. Numerical values are given as median and interquartile range (Q1–Q3).

RR values of CMW based on HPLC analysis of dialysate samples were strongly correlated with the optically estimated RR values (r = 0.958, *p* < 0.001) and similar (Table 3). Likewise, RR values of CMW based on HPLC analysis of serum samples were strongly correlated with spent dialysate-based optically estimated RR values (r = 0.939, *p* < 0.001) and similar (*p* = 0.347). Whereas the correlation was somewhat weaker between HPLC based and optically estimated CMW for TRS (r = 0.792, *p* < 0.001) and TAC (r = 0.717, *p* < 0.001).

## Discussion

Previous research has established that CMW is an endogenous metabolite, and a highly potential biomarker related to the health outcomes and CKD progression in earlier stages of CKD patients prior to ESKD. To our knowledge, CMW levels in ESKD patients and its hemodialytic removal have not been previously studied, although CMW has a potential intrinsic toxicity, and the concentration of CMW has been shown to rise in CKD patients. In this study, we filled this gap, and additionally created an optics-based predictive model for estimating hemodialytic removal and TAC of CMW non-invasively from spectrophotometric analysis of spent dialysate.

The main findings of this study were: (1) predialysis serum CMW concentrations of studied ESKD patients were presumably over 10 times higher compared to previously published CMW levels of healthy subjects; (2) in comparison to traditional maintenance HD adequacy marker-molecule urea, CMW is removed more efficiently (RR higher more than 6.7 percentage points) during HD; (3) CMW concentration in spent dialysate can be estimated optically with good accuracy (4) spent dialysate based optical monitoring allows to evaluate hemodialytic removal of CMW from patients’ blood and TAC of patients.

In the present study, the concentration of CMW in the collected samples was determined with a HPLC method earlier described by Arund et al*.* (2016) for analyzing the main fluorescent solutes, which are removed from ESKD patients’ blood into dialysis solution during HD^29^. Besides other identified tryptophan metabolites, Arund et al. found an unidentified fluorophore that was a significant constituent of the fluorescence signal (excitation: 280 nm; emission 360 nm) of spent dialysate samples, with a relative contribution to the fluorescence signal ranging between 2 and 16% in the analyzed spent dialysate samples^29^. The researchers hypothesized this compound, labeled as Unknown 1, to be glycoconjugate of tryptophan^29^. Here, we confirm that this previously unidentified uremic retention solute is CMW (Fig. 1) based on the comparison of mass spectra (Supplementary Fig. S1), fluorescence spectra and retention time of reference substance to that of CMW peak on chromatograms. Concerning HPLC analysis, a very good symmetry of the CMW peak on the chromatogram (Fig. 1) and full separation from the peaks of other fluorescent metabolites present in dialysate and serum was achieved. In detail, peak purity was assessed based on the absorbance and fluorescence spectra of the peak, confirming no co-elution of key analytes and high specificity of used HPLC method to separate CMW from other metabolites with no co-elution of contaminants in the peak. No decomposition of the CMW in samples was observed during the sample storage at 4 °C over 100 h, with peak areas being unchanged (relative standard deviation of peak areas < 0.93% based on samples of 3 patients).

Prior research has shown that average (± SD) plasma concentration of CMW in healthy control subjects (eGFR 104.01 ± 5.7 mL/min /1.73 m^2^) was 0.26 ± 0.05 µmol/L in the Qatar Metabolomics Study on Diabetes^40^, and 0.23 ± 0.05 µmol/L in serum of subjects (eGFR 114.1 ± 29.6 mL/min/1.73 m^2^) in a Japanese study^26^ focusing on diagnostic value of CMW concentration, respectively. In this analysis, the median (interquartile range) concentration of CMW in serum samples of ESKD patients was 2.78 (2.20–3.22) μmol/L prior to start of dialysis, which is presumably > 10 times higher than the average normal concentration in healthy controls. This is comparable to the estimated 13–16 fold increase of CMW in ESKD patients relative to healthy subjects reported by Tanaka et al*.* using relative amounts of metabolites^5^. Moreover, compared to CKD patients with eGFR of 50.9 ± 17.6 mL/min/1.73 m^2^ and average CMW concentration 0.72 ± 0.28 µmol/L in plasma^40^, CMW levels in dialysis patients in our study had raised further. As a limitation of this study, it was not possible to compare the relationships of CMW levels in ESKD patients and healthy individuals using the same method under identical conditions, as this study focused only on ESKD patients. In future studies, healthy subjects should be included as a control group to eliminate possible bias between the HPLC method of this study and methods used in prior research to improve the accuracy of the comparison of CMW levels in ESKD patients and healthy controls. However, to decrease the levels of CMW in ESKD patients, their intestinal and metabolic generation should be limited and extracorporeal removal enhanced like for other uremic solutes ^41^.

Our analysis shows that CMW is removed more efficiently during HD in relation to urea, a standard marker molecule of dialysis adequacy, (Fig. 2), with the RR of CMW being higher relative to RR of urea (> 6.7%) for all studied modalities (*p* < 0.001). Furthermore, hemodialytic removal of urea and CMW were considerably affected by treatment settings. In case of Low Flux HD with relatively low blood and dialysate flow rates, the average (± SD) RR was 66.4 ± 8.9% for CMW and 58.9 ± 7.0% for urea, which increased (*p* < 0.001) to 84.8 ± 6.1% and 78.1 ± 5.8% with the change to the most efficient HDF (High HDF), respectively (Supplementary Fig. S2). These results imply that removal characteristics of CMW are comparable to those of small water-soluble compounds, according to the classification proposed by EUTox in which uremic toxins are categorized into three groups: small water-soluble compounds, middle molecules and protein-bound solutes^4^. In contrast to the hydrophobic tryptophan, which is protein-bound solute^28^, CMW is more water soluble due to the polar mannose residue, based on HPLC analysis (Fig. 1), which showed that retention time of CMW was 24% shorter than that of tryptophan using hydrophobic chromatography column. Efficient extracorporeal removal and renal filtration^25^ of CMW may arise therefore from good water solubility of CMW. Besides, as the extent of post-dialytic rebound was in comparable magnitude for both CMW and urea (*p* = 0.692), CMW may have minimal resistance to intercompartmental shifts, similar to urea^42^.

Traditionally, HD adequacy has been assessed by small water-soluble compounds clearance and quantified by Kt/V_urea_ or its simplified form (RR of urea), based on analysis of blood samples^43^. Whereas analysis of spent dialysate using measurement of UV absorption has been proven to be reliable, non-invasive alternative for Kt/V determination^37,44^, which is used in haemodialysis machines for real-time dialysis adequacy monitoring on-line^45,46^. Also, optical methods have been developed for monitoring removal of characteristic biomarkers of uremic toxins with different physicochemical properties and dialytic removal patterns, such as protein bound indoxyl^29,47^, middle molecule beta-2-microglobulin^47,48^ and advanced glycation end product pentosidine^49^ among others, which may facilitate personalizing HD prescriptions^50^.

To estimate intradialytic removal and levels of CMW, an optics-based predictive model was created using stepwise linear regression in this study, which included three variables: fluorescence at Ex250Em380 and Ex260Em349, and UV absorbance at 295. A high accuracy (BIAS ± SE) and strong correlation between HPLC determined and model estimated CMW concentration in spent dialysate were achieved for both calibration and validation group, 0.00 ± 0.07 μmol/L (r = 0.960, *p* < 0.001) and − 0.02 ± 0.07 μmol/L (r = 0.939, *p* < 0.001), respectively. Although spent dialysate consists of individual fluorophores, which fluorescence spectra tend to partially overlap^29,51^ with CMW (Supplementary Fig. S4), the results presented here (Fig. 4) suggest that it is possible to evaluate concentration of CMW with relatively high accuracy using combination of wavelengths of fluorescence and UV absorbance. Additionally, it can be deduced that fluorescence of spent dialysate at excitation of 280 nm and emission at 350 nm can be partly attributed to CMW, a specific region of fluorescence (left shoulder of the main fluorescence peak of spent dialysate) for which fluorophores causing the signal remained unidentified previously^29,51^.

The developed model enabled to evaluate CMW-based dialysis adequacy parameters, such as RR, TRS and TAC non-invasively without blood sampling as CMW concentrations in blood can be determined from spent dialysate concentrations (Fig. 3). The results showed (Table 3) that optically assessed spent dialysate-based RRs of CMW were similar and strongly correlated to HPLC determined spent dialysate- and serum-based RRs (r = 0.958, and r = 0.939, respectively). While for TRS and TAC estimation, the correlation between the HPLC determined and optically estimated values were weaker (r = 0.792 (*p* < 0.001), and r = 0.717 (*p* < 0.001), respectively). The weaker correlation seen between blood and dialysate-based values can be partly attributed to the different sampling times of blood and spent dialysate samples in the beginning of treatments. Namely, spent dialysate samples were taken 7 min after the start of dialysis procedure and blood samples predialysis, which can cause divergence between serum and spent dialysate-based TAC values as described earlier^52^ for solutes with double pool kinetics or small distribution volume that are removed rapidly. Moreover, it is expectable that RR was most precisely estimated clinical parameter, as RR is a relative measure, which is less influenced by the possible prediction error of the developed model caused by other fluorophores and chromophores as these can have similar removal efficiency^29,53^. In future, alternative data analysis approach could be used instead of stepwise linear regression to combine and transform available variables to develop a more precise model using larger cohort and dataset. Especially, considering that the developed multiparametric linear model can be sensitive to fluorescence peak shifting that is related to the composition of spent dialysate, which could alter the relationship between the excitation–emission wavelength and concentration of CMW.

Clinically, optical monitoring of CMW removal or TAC could be used to determine whether the previously seen relationship between CMW concentration and clinically relevant outcomes in earlier stages of CKD patients prior to ESKD, such as residual kidney function decline and mortality, remains valid in ESKD patients. The pathophysiological effect of CMW remains unidentified so far and additional experimental studies should be undertaken to elaborate whether CMW has causal role in CKD progression or if it is a non-toxic marker of solute retention, which concentration rises as residual kidney function declines^25^. Considering this, it would be interesting to investigate whether effective removal of CMW during HD improves clinical outcomes of ESKD patients and if CMW could be additional uremic solute marker to be monitored during HD^50^. Regardless of potential uremic toxicity of CMW, CMW has been shown to be strongly correlated to clinical outcomes and has a potential to improve management of CKD patients independently or in combination with other biomarkers, as biomarkers do not need to be causally involved in the disease process to predict the risk of future outcomes^7,13,14,54^. With this in mind, CMW may be useful as a marker for preserving and monitoring residual kidney function of ESKD patients, which remains a complicated task^55,56^.

In summary, this study was set out to determine the levels of CMW in ESKD patients and its removal characteristics, assess the possibility for optical estimation of CMW concentrations in spent dialysate and dialysis adequacy parameters without blood sampling. We identified that CMW concentrations in ESKD patients may be over 10 times higher compared to CMW concentrations in healthy control subjects as reported in previous studies. The removal of CMW during HD is characteristic to small water-soluble uremic solutes and CMW is removed with higher efficiency compared to urea by dialysis treatment. The results show that the removal and levels of CMW can be monitored based on spectrophoto- and spectrofluorimetric analysis of spent dialysate, which makes it possible to evaluate CMW-based HD adequacy parameters, such as RR, TRS and TAC without blood sampling. To conclude, this study offers an alternative method for determination of CMW based on optical measurements, which has a potential to improve clinical management of ESKD patients.

## Electronic supplementary material

Below is the link to the electronic supplementary material.

Supplementary Material 1

## Data Availability

The data that support the findings of this study are available from Optofluid Technologies OÜ but restrictions apply to the availability of these data, which were used under license for the current study, and so are not publicly available. Data are however available from the corresponding author upon reasonable request and with permission of Optofluid Technologies OÜ.
