# Supplementary material for: Intradialytic optical assessment of C-mannosyl tryptophan removal using spent dialysate
Source: Sci Rep. 2025 Jun 12;15:20052. doi: 10.1038/s41598-025-01844-z (PMC12162850; doi:10.1038/s41598-025-01844-z)
Supplement: Supplementary file 1 — Supplementary Material 1 [file 41598_2025_1844_MOESM1_ESM.pdf]

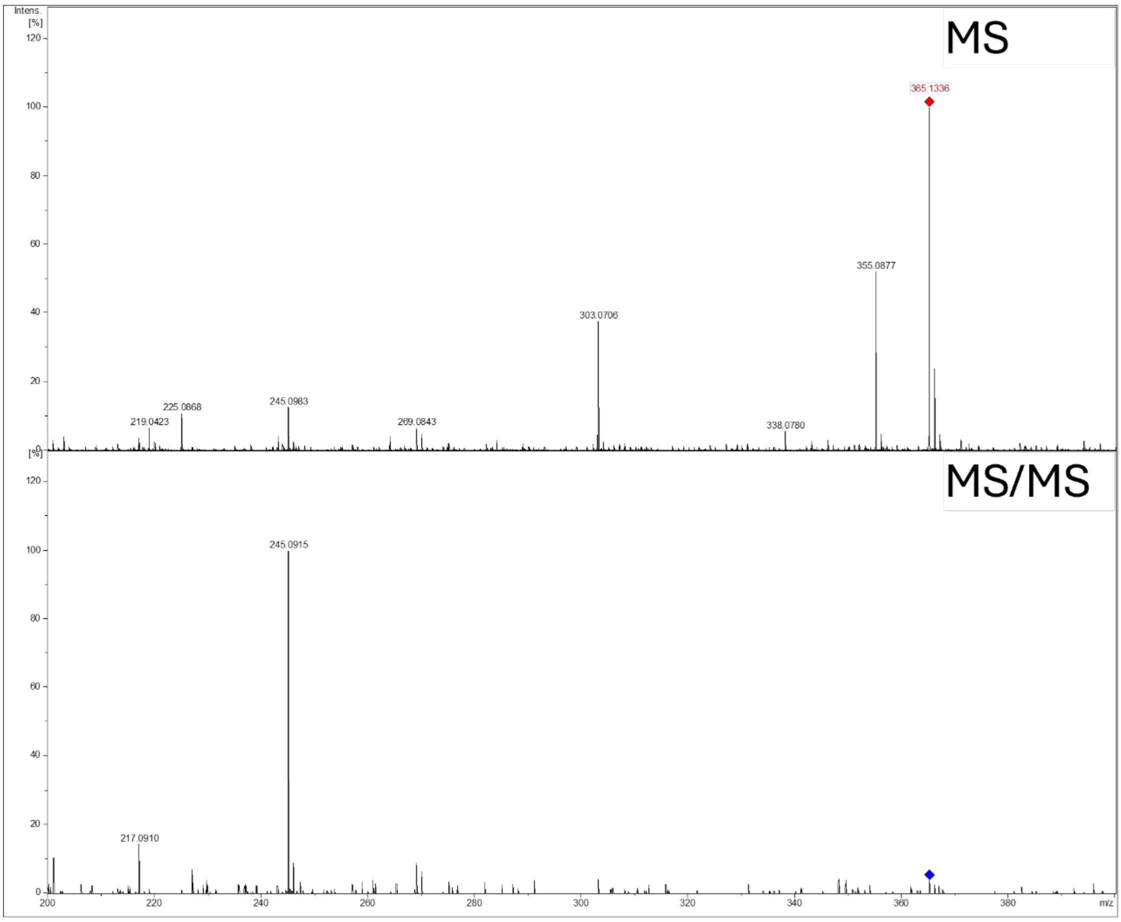


**Supplementary Figure S1.** Mass spectrum of the peak (MS) with retention time of 20.01 min on the chromatogram of spent dialysate sample (Figure 1) and product ion spectrum (MS/MS) of the precursor ion ($m/z^{-1}$ 365.13) with the fragment ion of $m/z^{-1}$ 245.09 characteristic for C-mannosyl tryptophan as reported earlier by Gutsche et al.[1] & Yamamoto et al[2].

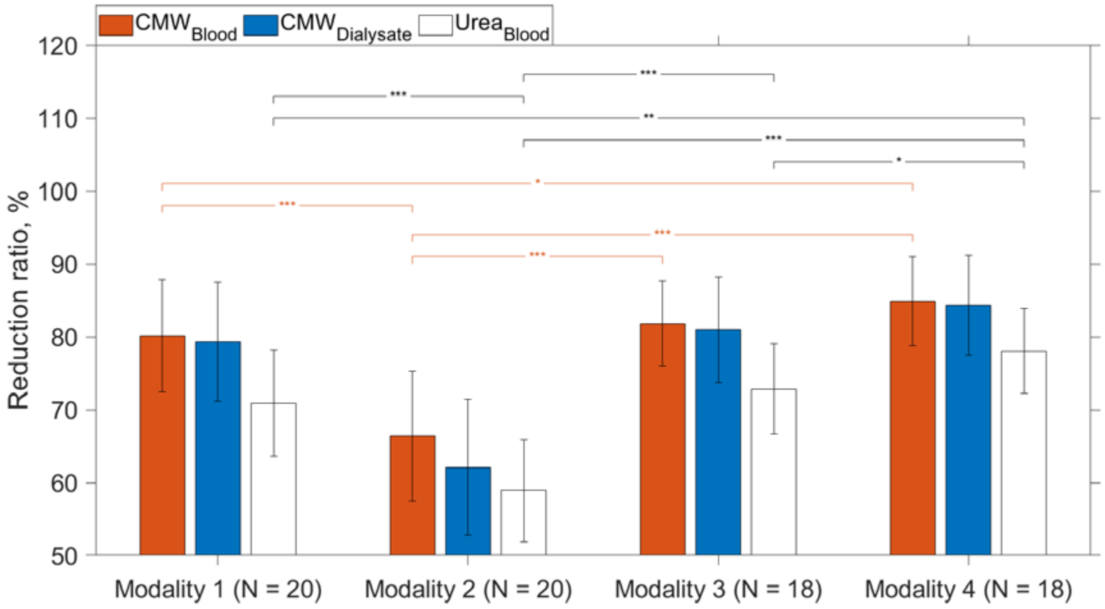


**Supplementary Figure S2.** Reduction ratios (Mean ± SD) of C-mannosyl tryptophan (CMW) and urea for different dialysis modalities (see Table 2 in Methods section) calculated based on serum and spent dialysate samples collected from the start and end (240 min) of the dialysis sessions; inter-modality statistical differences, evaluated using unpaired two-tailed t-tests, are denoted as follows: *** ($p < 0.001$), ** ($p < 0.01$), * ($p < 0.05$).

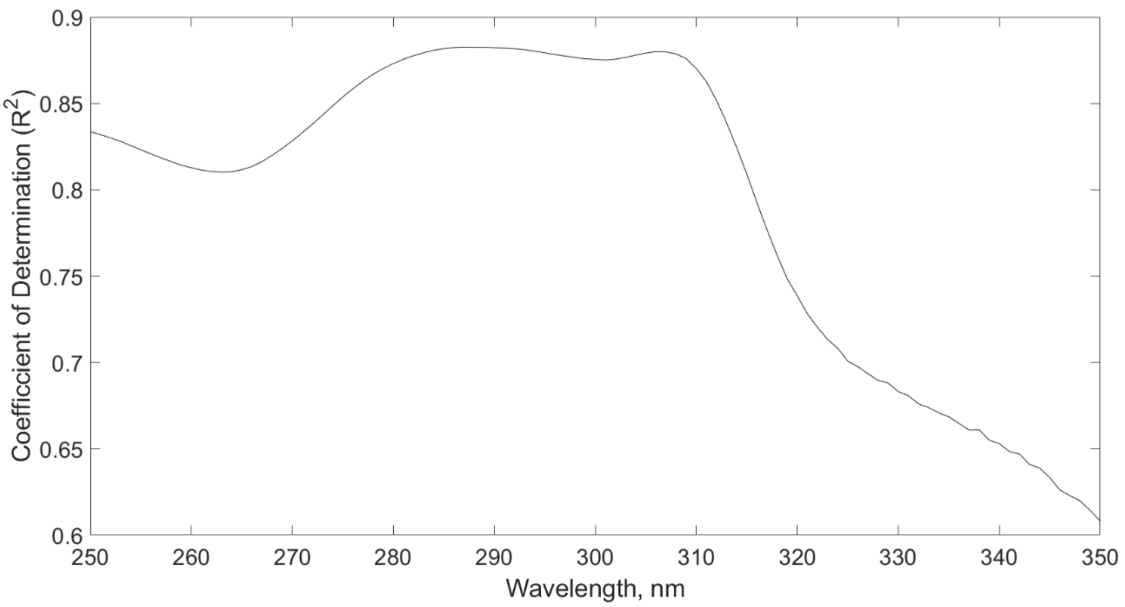


**Supplementary Figure S3.** Correlation between concentration of C-mannosyl tryptophan and UV absorbance of spent dialysate samples. UV absorption spectra were recorded with sampling interval of 1 nm (spectral bandwidth of 2 nm) and quartz cuvette with optical path length of 5 mm using pure dialysis solution as a reference.

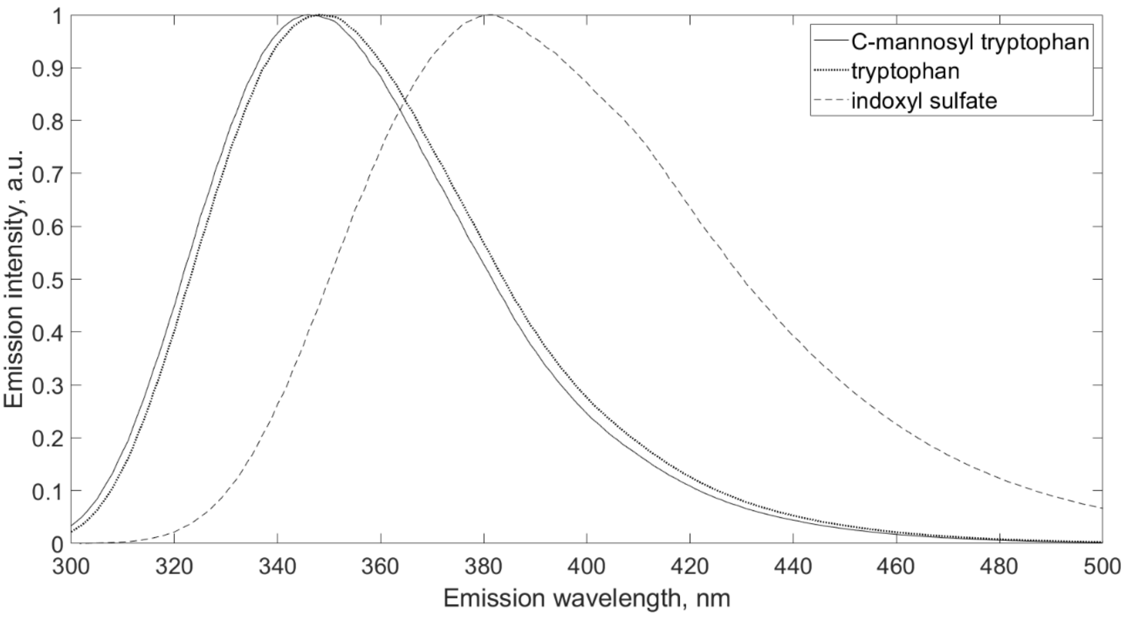


**Supplementary Figure S4.** Fluorescence spectra of C-mannosyl tryptophan, tryptophan and indoxyl sulfate at excitation 280 nm, normalized by the maximal value of each emission spectrum (346 nm, 348 nm and 381 nm, respectively). The solutes were dissolved in type I ultrapure water and fluorescence spectra were recorded with RF-6000 spectrofluorometer (Shimadzu, Kyoto, Japan) with excitation and emission bandwidth of 5 nm.

## References:

1. Gutsche, B., Grun, C., Scheutzow, D. & Herderich, M. Tryptophan glycoconjugates in food and human urine. *Biochem. J.* **343 Pt 1**, 11–9. http://www.ncbi.nlm.nih.gov/pubmed/10493906 (1999).

2. Yamamoto, M., Pinto-Sanchez, M. I., Bercik, P. & Britz-McKibbin, P. Metabolomics reveals elevated urinary excretion of collagen degradation and epithelial cell turnover products in irritable bowel syndrome patients. *Metabolomics* **15**, 82. https://doi.org/10.1007/s11306-019-1543-0 (2019).
